# Hardy type inequalities in $L^{p}$ with sharp remainders

**DOI:** 10.1186/s13660-016-1271-1

**Published:** 2017-01-03

**Authors:** Norisuke Ioku, Michinori Ishiwata, Tohru Ozawa

**Affiliations:** 1 Graduate School of Science and Engineering, Ehime University, Ehime, 790-8577 Japan; 2 Department of Systems Innovation, Graduate School of Engineering Science, Osaka University, Osaka, 560-8531 Japan; 3 Department of Applied Physics, Waseda University, Tokyo, 169-8555 Japan

**Keywords:** 26D10, 26D15, 46E35, Hardy’s inequalities, remainders

## Abstract

Sharp remainder terms are explicitly given on the standard Hardy inequalities in $L^{p}(\mathbb {R}^{n})$ with $1< p< n$. Those remainder terms provide a direct and exact understanding of Hardy type inequalities in the framework of equalities as well as of the nonexistence of nontrivial extremals.

## Results and discussion

The following Hardy inequalities are now well known:

1.1 $$\begin{aligned}& \biggl( \int_{0}^{\infty}x^{-r-1}\biggl\vert \int_{0}^{x}f(y)\,dy\biggr\vert ^{p} \biggr)^{\frac{1}{p}} \leq \frac{p}{r} \biggl( \int_{0}^{\infty}x^{p-r-1}\bigl|f(x)\bigr|^{p} \,dx \biggr)^{\frac{1}{p}}, \end{aligned}$$

1.2 $$\begin{aligned}& \biggl( \int_{0}^{\infty}x^{r-1}\biggl\vert \int_{x}^{\infty}f(y)\,dy\biggr\vert ^{p} \biggr)^{\frac{1}{p}} \leq \frac{p}{r} \biggl( \int_{0}^{\infty}x^{p+r-1}\bigl|f(x)\bigr|^{p} \,dx \biggr)^{\frac{1}{p}}, \end{aligned}$$

where $1\leq p<\infty$, $r>0$, and *f* is a real-valued measurable function on $(0,\infty)$,

1.3 $$ \biggl\Vert \frac{f}{|x|}\biggr\Vert _{L^{p}(\mathbb {R}^{n})} \leq \frac{p}{n-p} \biggl\Vert \frac{x}{|x|}\cdot\nabla f\biggr\Vert _{L^{p}(\mathbb {R}^{n})}, $$

where $1\leq p< n$ and $f\in W_{p}^{1}(\mathbb {R}^{n})$ (see [1, 2] for instance).

We revisit this famous inequality. Particularly, we present equalities which fill the gaps between the right- and left-hand sides of (1.1)-(1.3) with explicit remainder terms for $p>1$. Those equalities yield (1.1)-(1.3) by dropping remainder terms. Moreover, we give a characterization of functions which leads to vanishing remainders. The study of the Hardy inequalities which is based on the viewpoint of the equality leads to a direct and explicit understanding of the Hardy type inequalities as well as of the nonexistence of nontrivial extremals.

To state our main theorems, we introduce some necessary notation. In this paper, we deal with real-valued functions and we argue with sufficiently smooth functions with compact support in $\mathbb {R}^{n}\setminus\{0\}$ so that the standard density argument goes through. Let us introduce

1.4 $$\begin{aligned}& R_{p}(\xi,\eta) = \biggl(\frac{1}{p}|\eta|^{p}+ \frac{1}{p'}|\xi|^{p}-|\xi|^{p-2}\xi\eta \biggr) \big/|\xi- \eta|^{2}\quad\mbox{if }\xi\neq\eta, \end{aligned}$$

1.5 $$\begin{aligned}& R_{p}(\xi,\xi) = \frac{p-1}{2}|\xi|^{p-2}, \end{aligned}$$

for $p>1$ and *ξ*, $\eta\in \mathbb {R}$, where $1/p'=1-1/p$ and $R_{p}(\xi,\xi)$ makes sense only if $p\geq2$ and if $p<2$ and $\xi\neq0$. In other words, $R_{p}: (\xi,\eta)\mapsto R_{p}(\xi,\eta)$ is well defined on $\mathbb {R}\times \mathbb {R}$ if $p\geq2$ and on $(\mathbb {R}\times \mathbb {R})\setminus\{(0,0)\}$ if $1< p<2$. For *p* with $1\leq p\leq\infty$, the Banach space which consists of *p*th integrable Lebesgue measurable functions is denoted by $L^{p}(\Omega)$. The norm of it is also denoted by $\|\cdot\|_{L^{p}}$ or $\|\cdot\|_{p}$ if it does not cause confusion. The Sobolev space of order one introduced by $L^{p}$ is denoted $W^{1}_{p}=W^{1}_{p}(\mathbb {R}^{n})$ for $1\leq p<\infty$.

The basic properties of $R_{p}$ are summarized in the following proposition.

### Proposition 1

*Let* $p\in \mathbb {R}$ *satisfy* $p>1$. *Then* $R_{p}$ *satisfies the following properties*:

(1) $R_{p}$ *has the integral representation*

1.6 $$ R_{p}(\xi,\eta)=(p-1) \int_{0}^{1} \bigl\vert \theta\xi+(1-\theta)\eta \bigr\vert ^{p-2}\theta \,d\theta. $$

(2) $R_{p}$ *satisfies the estimates*

$$\begin{aligned}& R_{p}(\xi,\eta) \leq \textstyle\begin{cases} \frac{p-1}{2} (\vert \xi \vert \lor \vert \eta \vert )^{p-2}& \textit{if }p\geq 2,\\ \frac{p-1}{2} (\vert \xi \vert \land \vert \eta \vert )^{p-2}&\textit{if }p< 2, \end{cases}\displaystyle \\& R_{p}(\xi,\eta) \geq \textstyle\begin{cases} \frac{p-1}{2} (\vert \xi \vert \land \vert \eta \vert )^{p-2}&\textit{if }p\geq 2,\\ \frac{p-1}{2} (\vert \xi \vert \lor \vert \eta \vert )^{p-2}&\textit{if }p< 2, \end{cases}\displaystyle \end{aligned}$$

*where* $a\lor b=\max(a,b)$ *and* $a\land b=\min(a,b)$ *for* *a*, $b\geq0$.

(3) *Let* $p>2$ *and let* *ξ*, $\eta\in \mathbb {R}$. *Then* $R_{p}(\xi,\eta)=0$ *if and only if* $\xi=\eta=0$.

(4) *Let* $p\leq2$ *and let* *ξ*, $\eta\in \mathbb {R}\setminus\{0\}$. *Then* $R_{p}(\xi,\eta)>0$.

(5) $R_{2}(\xi,\eta)=\frac{1}{2}$ *for all* *ξ*, $\eta\in \mathbb {R}$.

We now state our main results.

### Theorem 1

*Let* *n* *and* *p* *satisfy* $1< p< n$. *Then the equality*

1.7 $$\begin{aligned} \biggl\Vert \frac{f}{|x|}\biggr\Vert _{L^{p}(\mathbb {R}^{n})}^{p} =& \biggl(\frac{p}{n-p} \biggr)^{p} \biggl\Vert \frac{x}{|x|} \cdot\nabla f\biggr\Vert _{L^{p}(\mathbb {R}^{n})}^{p} \\ &{}- p \int_{\mathbb {R}^{n}}R_{p} \biggl(\frac{1}{|x|}f,- \frac{p}{n-p}\frac{x}{|x|}\cdot\nabla f \biggr) \biggl\vert \frac{p}{n-p}\frac{x}{|x|}\cdot\nabla f+\frac{1}{|x|}f\biggr\vert ^{2}\,dx \end{aligned}$$

*holds for all* $f\in W^{1}_{p}(\mathbb {R}^{n})$. *If the second term on the right hand side of* (1.7) *vanishes*, *then the left*-*hand side of* (1.7) *is finite if and only if* $f=0$.

### Remark 1

In fact, we prove that if the second term on the right hand side of (1.7) vanishes, then there exists a function $\varphi: S^{n-1}\to \mathbb {R}$ on the unit sphere $S^{n-1}$ such that

1.8 $$ f(x)=|x|^{-\frac{n-p}{p}}\varphi \biggl(\frac{x}{|x|} \biggr) $$

almost everywhere in $\mathbb {R}^{n}\setminus\{0\}$. In this case,

1.9 $$ \frac{|f(x)|^{p}}{|x|^{p}} = \frac{\vert \varphi (\frac{x}{|x|} )\vert ^{p}}{|x|^{n}} $$

and the left-hand side of (1.7) is finite if and only if $\varphi=f=0$.

### Remark 2

The special case $p=2$ is studied in [3].

### Theorem 2

*Let* *p* *and* *r* *satisfy* $1< p<\infty$ *and* $r>0$. *Then*:

(1) *The equality*

1.10 $$\begin{aligned} \int_{0}^{\infty}x^{-r-1}\biggl\vert \int_{0}^{x}f(y)\,dy\biggr\vert ^{p}\,dx =& \biggl(\frac{p}{r} \biggr)^{p} \int_{0}^{\infty}x^{p-r-1}\bigl\vert f(x)\bigr\vert ^{p}\,dx \\ & {}-p \int_{0}^{\infty}R_{p} \biggl(x^{-\frac{r+1}{p}} \int_{0}^{x}f, \frac{p}{r}x^{1-\frac{r+1}{p}}f \biggr) \\ &{}\times \biggl\vert x^{-\frac{r+1}{p}} \biggl( \frac{p}{r}xf- \int_{0}^{x}f \biggr) \biggr\vert ^{2} \,dx \end{aligned}$$

*holds for all real*-*valued measurable functions on* $(0,\infty)$ *with* $xf\in L^{p}(0,\infty; x^{-r-1}\,dx)$. *Moreover*, *there exists* $c\in \mathbb {R}$ *which satisfies*, *for almost everywhere* $x\in(0,\infty)$,

1.11 $$ f(x)=cx^{\frac{r}{p}-1} $$

*when the last term in the right hand side of* (1.10) *equals zero*. *In this case*,

1.12 $$ x^{-r-1}\biggl\vert \int_{0}^{x}f(y)\,dy\biggr\vert ^{p}=|c|^{p} \biggl(\frac{p}{r} \biggr)^{p}x^{-1} $$

*and the left*-*hand side of* (1.10) *is finite if and only if* $c=0$.

(2) *The equality*

1.13 $$\begin{aligned}& \int_{0}^{\infty}x^{r-1}\biggl\vert \int_{x}^{\infty}f(y)\,dy\biggr\vert ^{p} \,dx \\& \quad= \biggl(\frac{p}{r} \biggr)^{p} \int_{0}^{\infty}x^{p+r-1}\bigl\vert f(x)\bigr\vert ^{p}\,dx \\& \qquad {}-p \int_{0}^{\infty}R_{p} \biggl(x^{\frac{r-1}{p}} \int_{x}^{\infty}f, \frac{p}{r}x^{1+\frac{r-1}{p}}f \biggr) \biggl\vert x^{\frac{r-1}{p}} \biggl(\frac{p}{r}xf- \int_{x}^{\infty}f \biggr) \biggr\vert ^{2} \,dx \end{aligned}$$

*holds for all real*-*valued measurable functions on* $(0,\infty)$ *with* $xf\in L^{p}(0,\infty; x^{r-1}\,dx)$. *Moreover*, *there exists* $c\in \mathbb {R}$ *which satisfies*, *for almost everywhere* $x\in(0,\infty)$,

1.14 $$ f(x)=cx^{-\frac{r}{p}-1} $$

*provided that the last term in the right hand side of* (1.13) *vanishes*. *In this case*,

1.15 $$ x^{r-1}\biggl\vert \int_{x}^{\infty}f(y)\,dy\biggr\vert ^{p} = |c|^{p} \biggl(\frac{p}{r} \biggr)^{p}x^{-1} $$

*and the left*-*hand side of* (1.13) *is finite if and only if* $c=0$.

### Remark 3

The special case $p=2$ is studied in [3].

We prove the theorems in subsequent sections. The first step of the proof is the same as the standard one. We need the following identity:

1.16 $$ \int\frac{|f(x)|^{p}}{|x|^{p}}\,dx = -\frac{p}{n-p} \int\frac{|f(x)|^{p-2}f(x)}{|x|^{p-1}}\frac {x}{|x|}\cdot\nabla f(x)\,dx, $$

which holds for all $f\in C_{0}^{\infty}(\mathbb {R}^{n})$, provided $1< p< n$. It can be obtained expressing the integral on the left-hand side by means of the spherical coordinates and using the integration by parts (*cf.* [4], Proof of Theorem 1.1).

Equation (1.16) together with the Hölder inequality with $1/p+1/p'=1$, $1\leq p<\infty$, implies (1.3). In this sense, the standard method depends upon duality. In this paper, we adopt a different view. We rewrite (1.16) in the form

1.17 $$ \int|u|^{p}\,dx= \int|u|^{p-2}uv\,dx $$

with $u=\frac{f}{|x|}$ and $v=-\frac{p}{n-p}\frac{x}{|x|}\cdot \nabla f$ and modify (1.17) as

1.18 $$ \int \bigl( \vert u\vert ^{p}-\vert u\vert ^{p-2}uv\bigr)\,dx=0. $$

Now the equality (1.18) can be understood as representing a cancelation as well as an oscillation or an orthogonality. This point of view for equation (1.18) can be stated in the following way.

### Lemma 1

*Let* $L^{p}(\Omega,\mu)$ *with* $1< p<\infty$ *be the Banach space of* *pth integrable real*-*valued functions on a measure space* $(\Omega,\mu)$ *endowed with a norm* $\|\cdot\|_{p}$. *Then the following three assertions are equivalent for any* *u*, $v\in L^{p}(\Omega,\mu)$:

1. *We have*
  1.19 $$ \Vert u\Vert _{p}^{p}= \int_{\Omega} \vert u\vert ^{p-2}uv\,d\mu. $$
2. *We have*
  1.20 $$ \Vert u\Vert _{p}^{p}=\Vert v\Vert _{p}^{p}- \int_{\Omega} \bigl( \vert v\vert ^{p}+(p-1)\vert u \vert ^{p}-p\vert u\vert ^{p-2}uv \bigr)\,d\mu. $$
3. *We have*
  1.21 $$ \Vert u\Vert _{p}^{p}=\Vert v\Vert _{p}^{p}-p \int_{\Omega}R_{p}(u,v) \vert u-v\vert ^{2} \,d\mu. $$

### Proof of Lemma 1

The assertions are trivial for $u=v$. If $u\neq v$, then the relation

$$\begin{aligned}& \Vert v\Vert _{p}^{p}-\Vert u\Vert _{p}^{p}+p \int_{\Omega}\bigl(\vert u\vert ^{p}-\vert u\vert ^{p-2}uv\bigr)\,d\mu \\& \quad= \int_{\Omega} \bigl(\vert v\vert ^{p}+(p-1)\vert u \vert ^{p}-p\vert u\vert ^{p-2}uv\bigr)\,d\mu = p \int_{\Omega}R_{p}(u,v)\vert u-v\vert ^{2} \,d\mu \end{aligned}$$

immediately yields the conclusion. □

The subsequent sections are organized as follows. Proposition 1 will be proved in Section 2. Section 3 is devoted to the verification of Theorem 1. The proof of Theorem 2 is given in Section 4. There is a large literature on Hardy type inequalities and related subjects. See [1–32] and the references therein for instance.

## Proof of Proposition 1

First of all, we remark that $R_{2}(\xi,\eta)=1/2$, by definition. This proves Part (5) as well as Parts (1), (2), and (4) for $p=2$. By a direct calculation, (1.6) holds if $\xi=\eta$. Let $\xi\neq\eta$. We obtain

$$\begin{aligned}& \frac{1}{p}\vert \eta \vert ^{p}+\frac{1}{p'}\vert \xi \vert ^{p}-\vert \xi \vert ^{p-2}\xi\eta \\& \quad= \biggl(1-\frac{1}{p} \biggr) \bigl(\vert \xi \vert ^{p}-\vert \eta \vert ^{p}\bigr)-\eta\bigl(\vert \xi \vert ^{p-2}\xi -\vert \eta \vert ^{p-2} \eta\bigr) \\& \quad= (p-1) \int_{0}^{1}\bigl\vert \theta\xi+(1-\theta)\eta \bigr\vert ^{p-2}\bigl(\theta\xi +(1-\theta)\eta\bigr) \,d\theta(\xi- \eta) \\& \qquad {}-(p-1) \int_{0}^{1} \bigl\vert \theta\xi+(1-\theta)\eta \bigr\vert ^{p-2}\,d\theta\eta(\xi -\eta) \\& \quad= (p-1) \int_{0}^{1} \bigl\vert \theta\xi+(1-\theta)\eta \bigr\vert ^{p-2}\theta \,d\theta (\xi-\eta)^{2}, \end{aligned}$$

which yields (1.6). Then Part (2) follows immediately from Part (1). If $p>2$ and $R_{p}(\xi,\eta)=0$, then by the integral representation (1.6) we have $\theta\xi+(1-\theta)\eta=0$ for any *θ* with $0<\theta<1$. This implies $\xi=\eta=0$. If $p<2$ and $R_{p}(\xi,\eta)=0$, then $|\theta\xi+(1-\theta)\eta|=\infty$ for any *θ* with $0<\theta<1$, which is absurd. This proves Proposition 1.

## Proof of Theorem 1

By a standard density argument, it is enough to prove Theorem 1 for $f\in C_{0}^{\infty}(\mathbb {R}^{n})$. Applying (1.16), (1.7) is then a direct consequence of Lemma 1 with $u=\frac{f}{|x|}$ and $v=-\frac{p}{n-p}\frac{x}{|x|}\cdot\nabla f$. Now suppose that the second term on the right hand side of (1.7) vanishes. Then by Parts (3) and (4) of Proposition 1, we easily see that *f* satisfies the equation

$$\frac{p}{n-p}\frac{x}{|x|}\cdot\nabla f+\frac{1}{|x|}f=0, $$

which is equivalent to

$$\frac{x}{|x|}\cdot\nabla \bigl(|x|^{\frac{n-p}{p}}f \bigr)=0. $$

This implies (1.8), which in turn implies the rest of the statements of the theorem.

## Proof of Theorem 2

By integration by parts, we have

$$\int_{0}^{\infty}x^{-r-1}\biggl\vert \int_{0}^{x}f\biggr\vert ^{p}\,dx = \frac{p}{r} \int_{0}^{\infty}x^{-r}\biggl\vert \int_{0}^{x}f\biggr\vert ^{p-2} \int_{0}^{x}f\cdot f\,dx, $$

so that (1.10) follows from Lemma 1 by setting $u=x^{-\frac{r+1}{p}}\int_{0}^{x}f$ and $v=x^{1-\frac{r+1}{p}}f$. The rest of the statements of Part (1) follow if we notice that

$$\frac{p}{r}xf- \int_{0}^{x}f=\frac{p}{r}x^{1+\frac{p}{r}} \frac{d}{dx} \biggl(x^{-\frac{p}{r}} \int_{0}^{x} f \biggr). $$

Part (2) follows by the same argument.

## Conclusions

In this paper, we examined the sharp remainder terms of the Hardy inequality for $L^{p}$-functions. From these sharp remainder terms, we can derive several consequences including the explicit form of the extremal function for the inequality which reveals the nature of the nonexistence of extremals in the $L^{p}$-setting. Our analysis only requires some elementary calculus with some insight in the structure of the remainder term and is also applicable to other critical type inequalities such as the Hardy inequalities in $L^{n}$.
